# Mapping benefit, risk, and opportunity in PAD4 inhibition

**DOI:** 10.3389/fimmu.2026.1769421

**Published:** 2026-02-26

**Authors:** Caio Santos Bonilha, Flavio Protasio Veras

**Affiliations:** 1 Institute of Infection, Immunity and Inflammation, University of Glasgow, Glasgow, United Kingdom; 2 Institute of Biomedical Sciences, Federal University of Alfenas, Alfenas, Brazil; 3 São Carlos Institute of Physics, University of São Paulo, São Paulo, Brazil

**Keywords:** immune regulation, neutrophil extracellular traps, neutrophils, protein citrullination, translation immunology

## Abstract

Peptidylarginine deiminase 4 (PAD4) is increasingly targeted to modulate inflammatory pathology, yet its inhibition produces biological effects that extend beyond the processes it was originally designed to suppress. While PAD4 targeting has largely been pursued to limit neutrophil extracellular trap (NET) formation, accumulating data indicate that PAD4 activity also shapes immune regulation through citrullination of non-histone substrates, with consequences for antigen presentation, cytokine function, and adaptive immune activation. These broader effects introduce important considerations for translation, as PAD4 inhibition can simultaneously attenuate tissue-damaging inflammation and undermine protective host responses. In this review, we examine PAD4 targeting through a benefit–risk–opportunity framework that integrates enzymatic specificity, cellular context, and disease setting. We discuss how suppression of NET-driven pathology underlies therapeutic benefit in thrombo-inflammatory disease, how impaired control of microbial dissemination represents a central risk in infection, and how direct effects on dendritic- and T-cell-mediated responses may be leveraged in autoimmune contexts. Rather than reflecting unintended drug activity, many immune effects attributed to off-target inhibition arise from disruption of citrullination-dependent regulatory pathways. This perspective provides a mechanistic basis for selecting indications, designing combination strategies, and defining appropriate safety endpoints, supporting a more precise and context-aware approach to PAD4 targeting in immune-mediated disease.

## 1 Introduction

Peptidylarginine deiminase 4 (PAD4) has emerged as an important therapeutic target across a broad range of inflammatory diseases. This reflects its established role in histone citrullination, chromatin decondensation, and the formation of neutrophil extracellular traps (NETs), which are web-like DNA–protein structures released by activated neutrophils (1). NET-associated pathology has been reported in many conditions, such as severe infection, thrombosis, cancer, and autoimmune disease (1), supporting PAD4 inhibition as a strategy to limit excessive inflammation. The rapid expansion of PAD4-focused research, particularly during the COVID-19 pandemic, has accelerated the development of pharmacological inhibitors and generated substantial preclinical evidence suggesting therapeutic benefit (2–5). However, much of this progress has been guided by NET-focused readouts that implicitly frame PAD4 inhibition as a restricted intervention with localized and predictable effects.

Increasing evidence indicates that this perspective is incomplete. PAD4 activity extends beyond NET formation (NETosis) and contributes to immune regulation through mechanisms that include cytokine (6) and chemokine (7) modification, antigen availability (8), and coordination between innate and adaptive immune responses (9). In parallel, pharmacological inhibition of PAD4 introduces additional considerations related to isozyme cross-inhibition and chemical scaffold-associated liabilities (10). These effects are often described as off-target, yet many represent coherent biological outcomes of disrupting citrullination-dependent pathways rather than unintended drug effects. In this review, PAD4 inhibition is evaluated using a risk-mapping approach that integrates enzymatic specificity, immune context, and translational considerations to clarify when targeting is mechanistically justified, when risk predominates, and when broader immune effects may be therapeutically advantageous.

## 2 PAD4 activity across immune contexts

PAD4 operates as a context-dependent regulator of inflammatory immune responses, with activity that is closely coupled to immune activation rather than homeostatic function. In immune cells, PAD4 engagement is triggered by inflammatory cues associated with neutrophil activation, tissue damage, and cellular stress, situating its activity within acute immune responses. Through citrullination of nuclear and cytoplasmic substrates, PAD4 influences chromatin accessibility and transcriptional programs linked to inflammatory effector function, including hypoxia-responsive gene expression, regulation of p53-dependent transcriptional stability, and activation of viral transcriptional programs (11–13). In neutrophils, this activity supports NETosis, while in other immune contexts it contributes to regulation of gene expression and activation thresholds (1). Importantly, PAD4-dependent effects are typically observed under conditions in which the enzyme is catalytically engaged, meaning that its detectable impact is most readily apparent in immune environments characterized by heightened activation, where innate and adaptive responses coincide. This context dependence is central to interpreting how PAD4 inhibition alters immune responses, as the magnitude and nature of its effects are shaped by the immune state in which citrullination-dependent pathways are engaged.

Within this immune setting, PAD4 must be considered alongside other members of the PAD family, including PAD1, PAD2, PAD3, and PAD6, which differ in expression patterns, calcium sensitivity, and substrate preferences. Among these, PAD2 exhibits the greatest functional overlap with PAD4 in immune tissues and shares several substrates, including histones and transcriptional regulators (14–17). However, PAD2 differs in subcellular localization and tissue distribution, with broader expression across myeloid and lymphoid compartments as well as non-immune tissues (15, 18, 19). These similarities and differences have direct implications for pharmacological targeting, as many inhibitors developed to suppress PAD4 activity also inhibit PAD2 at concentrations commonly used in cellular and *in vivo* studies (20). Such isozyme cross-inhibition can blur mechanistic attribution, particularly when changes in gene regulation or immune activation are ascribed solely to PAD4 blockade.

The relevance of isozyme overlap becomes more apparent when considering the cellular distribution of PAD4 across the immune system. While neutrophils represent the most extensively studied context for PAD4 activity, PAD4 expression and function extend to additional immune and vascular cell types. Monocytes and dendritic cells (DCs) express PAD4, where it has been linked to chromatin remodeling, transcriptional regulation, and antigen presentation (8, 9, 21). PAD4 has been shown to localize to multiple subcellular compartments in monocytes, including cytosolic, organelle-associated, and cell surface pools that expand during differentiation into monocyte-derived DCs, supporting a role for PAD4 in antigen handling and intracellular immune coordination beyond the nucleus (22). In platelets and megakaryocytes, PAD4 connects citrullination to platelet activation, immunothrombosis, and coagulation pathways (23). Endothelial PAD4 activity has also been associated with inflammatory activation and barrier dysfunction under pathological conditions (24). Taken together, these observations indicate that PAD4 inhibition exerts coordinated effects across multiple cellular compartments, reinforcing the need to interpret its biological consequences beyond a neutrophil-centered framework.

## 3 Defining and characterizing PAD4 inhibition

The context-dependent activation and broad cellular distribution of PAD4 raise a fundamental question regarding how its inhibition should be defined in biological systems. On-target PAD4 inhibition implies effective suppression of its catalytic activity, resulting in reduced citrullination of relevant protein substrates under conditions in which the enzyme would normally be active. In experimental settings, this can be assessed using biochemical activity assays, measurements of substrate occupancy, or evaluation of global changes in the cellular citrullinome (16, 20). Each of these approaches provides useful but partial information, and none alone captures functional target engagement within complex immune environments. As a result, apparent PAD4 inhibition is frequently inferred indirectly, emphasizing the need for careful interpretation of downstream biological readouts.

This reliance on indirect assessment has contributed to the widespread use of NET-associated markers as surrogate indicators of PAD4 inhibition. Commonly used readouts include citrullinated histone H3, myeloperoxidase DNA complexes, and fluorescence-based imaging of extracellular chromatin. While experimentally convenient, these measures capture only a narrow subset of PAD4-dependent biology and are susceptible to confounding by alternative forms of cell death, including apoptosis and necrosis (25, 26). In addition, NET-associated markers do not account for PAD4 activity in non-neutrophil compartments, nor do they reflect citrullination events that influence immune regulation in the absence of overt chromatin release. Suppression of these markers may therefore provide an incomplete representation of the biological consequences of PAD4 inhibition.

Interpretation is further complicated by the diversity of experimental approaches used to inhibit PAD4 in immune settings. Compounds employed across studies differ in how broadly they affect immune cells, how sustained their effects are *in vivo*, and which inflammatory contexts they influence. Although these agents are commonly grouped together as PAD4 inhibitors, their use can lead to distinct immune outcomes, even when similar reductions in NET-associated markers are observed. This principle extends beyond small-molecule approaches, as antibody mediated modulation of PAD4 demonstrates that comparable suppression of commonly used readouts can emerge from distinct modes of regulatory interference with divergent immune consequences (27). In models of inflammation, differences in the mode and context of PAD4 modulation can shape the extent and distribution of immune modulation, influencing how neutrophil-driven pathology, vascular inflammation, or broader immune responses are altered. Consequently, comparable experimental readouts do not necessarily reflect equivalent immune effects. This variability limits direct comparison across studies and reinforces the need to interpret PAD4 inhibition in terms of immune context and phenotype, rather than assuming uniform consequences based solely on suppression of individual markers.

Selectivity represents an additional determinant of how PAD4 inhibition manifests at the cellular and organismal level. Many compounds exhibit partial cross-inhibition of PAD2 at concentrations required for cellular activity, reflecting structural similarities within the PAD family (14–17). Commonly used PAD4 inhibitors, including Cl-amidine, GSK484, and related chemical scaffolds, effectively suppress PAD4 catalytic activity in biochemical and preclinical models but differ in reversibility, exposure requirements, and isozyme selectivity (10). At concentrations necessary for sustained cellular or *in vivo* efficacy, these agents frequently engage PAD2 and broaden citrullination suppression (15), shaping both their biological impact and associated risk profile. In parallel, pharmacokinetic and pharmacodynamic factors, including plasma protein binding, tissue distribution, and dosing duration, shape the extent and persistence of target engagement *in vivo*. Exposure levels achieved in preclinical models may therefore exceed those predicted from *in vitro* assays, increasing the likelihood of isozyme cross-inhibition and scaffold-dependent non-specific effects (28). Together, these considerations underscore that PAD4 inhibition is not a singular intervention but a composite of enzymatic specificity, cellular context, and pharmacological behavior, providing a necessary framework for evaluating its benefits and risks across disease settings.

## 4 PAD4 targeting from benefit to risk to opportunity

### 4.1 On-target benefits and translational signals

Pharmacological suppression of PAD4 activity produces measurable biological effects in disease settings characterized by excessive citrullination and neutrophil activation. In infectious contexts, PAD4-dependent NETosis contributes to the restriction of microbial dissemination, while simultaneously amplifying tissue injury when dysregulated (1). Inhibition of PAD4 has therefore been associated with reduced inflammatory damage in models of severe infection, although this benefit is frequently accompanied by impaired pathogen clearance. Similar trade-offs are observed in viral infection, where PAD4 inhibition limits immunopathology driven by excessive neutrophil activation but may compromise antiviral defense under certain conditions (9, 29). These patterns indicate that PAD4 inhibition is most effective where inflammatory amplification dominates disease pathology, whereas its use might be less suitable in contexts that rely on intact antimicrobial immunity.

Beyond infection, PAD4 activity also shapes immune responses at the interface between inflammation and coagulation. PAD4-mediated citrullination promotes chromatin decondensation and extracellular DNA release, generating structures that support platelet activation, coagulation factor engagement, and thrombus stabilization. Experimental models of immunothrombosis and venous thrombosis show that disruption of PAD4 activity reduces thrombus burden and vascular inflammation without fully abolishing hemostatic function (30–32). In inflammatory settings such as severe sepsis, platelets have emerged as active drivers of immune amplification, with platelet-derived signals reinforcing NET formation and sustaining inflammatory feedback loops (23, 33). Within this context, PAD4-dependent NET scaffolds provide a mechanistic link between platelet activation, neutrophil effector function, and vascular inflammation. PAD4 activity has also been implicated in cancer-associated thrombosis and metastatic niche formation, where NETs facilitate tumor cell seeding and immune evasion (5, 34). Together, these observations position PAD4 inhibition as a mechanistically coherent strategy in settings where inflammatory and thrombotic programs converge, while underscoring the need to balance anti-thrombotic benefit against host defense across disease contexts with distinct benefit–risk profiles (**Table 1**).

**Table 1:** Context-dependent consequences of PAD4 inhibition across disease settings.

Disease context	Dominant mechanism	Main biological consequence	Translational interpretation	Representative recent reference
Microbial infection	PAD4-dependent NETosis during bacterial challenge	Reduced NET release and attenuated inflammatory tissue injury with impaired pathogen control	Context-dependent benefit with increased infection risk	(35)
Viral infection	Neutrophil-driven NET accumulation and inflammatory amplification	Reduced NET burden and inflammatory tissue damage with compromised antiviral immune responses	Narrow therapeutic window	(29)
Thrombo-inflammation and sepsis	NET–platelet interactions and NET scaffolding in venous thrombosis	Reduced NET-associated thrombus formation and altered platelet–NET dynamics	Favorable in venous thrombo-inflammatory settings with bleeding risk consideration	(33)
Cancer	PAD4-driven citrullination supporting tumor-associated inflammation	Reduced tumor growth and inflammatory progression following PAD4 antagonism	Mechanistically relevant tumor-intrinsic opportunity	(36)
Autoimmunity	PAD4-dependent NET activity and inflammatory tissue remodeling	Reduced inflammatory tissue damage and disease severity following PAD4 inhibition	Mechanistically favorable when immune dampening is acceptable	(37)
Adaptive immune regulation	Citrullination effects on antigen presentation and IL-2 production	Reduced T-cell priming and effector expansion	Risk in infection and opportunity in autoimmunity	(9)

### 4.2 Citrullination beyond histones

Although histone citrullination represents the most visible consequence of PAD4 activation, the enzyme modifies a broader repertoire of substrates that extend into core immune regulatory pathways. PAD4-mediated citrullination of cytokines and chemokines alters charge distribution and protein conformation, thereby influencing receptor binding, gradient formation, and signaling potency (6, 7). Modification of these mediators can reshape leukocyte recruitment, adjust the magnitude and duration of inflammatory responses, and influence the balance between immune amplification and resolution. As such, these effects can occur independently of NET release, positioning citrullination as a regulatory mechanism that operates even in the absence of overt chromatin extrusion.

Extending beyond soluble mediators, PAD4-dependent citrullination also influences immune regulation at the level of cellular activation and intercellular coordination. In antigen-presenting cells, altered citrullination affects chromatin accessibility and co-stimulatory signaling, constraining antigen presentation efficiency and downstream CD4 T-cell priming. This, in turn, is associated with reduced production of interleukin-2 (IL-2) and limited expansion of activated T-cell populations (9, 38). Together with reduced citrullination of inflammatory mediators, these effects illustrate how PAD4 inhibition modulates immune amplification through NET-independent pathways that can produce divergent functional outcomes depending on context. Through these combined effects, PAD4 inhibition links changes in protein citrullination to broader modulation of adaptive immune activation, providing a mechanistic bridge between molecular substrate modification and system-level immune outcomes.

### 4.3 System-level and molecular risks

The cumulative impact of altered citrullination across multiple substrates becomes evident at the system level, where PAD4 inhibition reshapes immune coordination rather than targeting a single effector pathway. NETs contribute to spatial organization of inflammation and communication between innate immune populations, and their attenuation can disrupt these processes beyond the site of pathology (1). When combined with reduced cytokine and chemokine citrullination, PAD4 inhibition can blunt immune cell recruitment, delay tissue repair, and alter inflammatory resolution (5–7, 39). These effects help explain the increased susceptibility to infection and impaired wound healing reported in experimental models involving PAD4 blockade (40, 41).

System-level risk is further shaped by pharmacological and molecular considerations. Partial cross-inhibition of PAD2 can influence transcriptional regulation and immune cell differentiation, compounding the effects of PAD4-specific blockade (15, 16). Together, these factors contribute to a broader erosion of citrullination-dependent immune coordination, increasing vulnerability to infection, impaired tissue repair, and dysregulated inflammatory resolution. Framed in this way, the risks associated with PAD4 inhibition reflect not only suppression of NETosis but also disruption of interconnected immune regulatory processes that operate across multiple cellular and tissue compartments.

### 4.4 Reframing risk as opportunity in autoimmunity

In autoimmune disease, mechanisms that underlie risk in infectious settings may instead align with therapeutic goals. Citrullinated proteins contribute to neoantigen generation and loss of tolerance in conditions such as rheumatoid arthritis (42, 43). By limiting citrullination across histone and non-histone substrates, PAD4 inhibition may reduce autoantigen availability, constrain T-cell priming, and dampen IL-2-dependent expansion of autoreactive T cells (9, 44). In this context, partial suppression of immune activation may interrupt self-sustaining inflammatory circuits that drive chronic disease.

**Figure 1:**
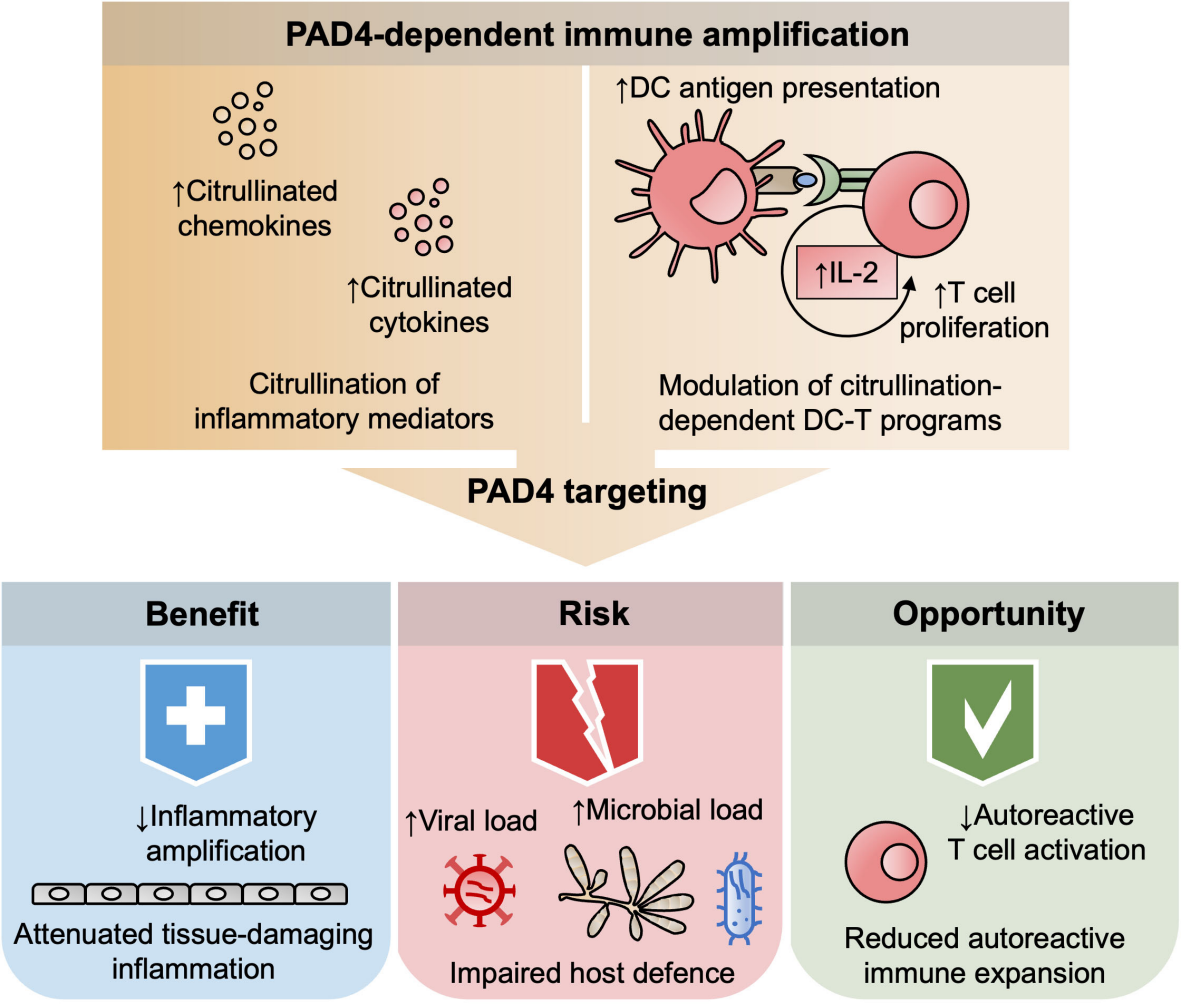
Established immune effects and emerging opportunities of PAD4 inhibition beyond NETosis. Schematic overview illustrating NET-independent immune consequences of PAD4 inhibition. The upper panel highlights well-established effects of reduced citrullination beyond histones, including altered modification of inflammatory mediators and modulation of citrullination-dependent dendritic cell–T cell programs, which together influence the magnitude of immune amplification. The lower panel depicts how these shared upstream effects can diverge into context-dependent outcomes. Established benefits include reduced inflammatory amplification and limitation of tissue-damaging immune responses, whereas recognized risks involve impaired host defense associated with increased microbial or viral burden. In parallel, emerging opportunities are illustrated by the potential to dampen autoreactive T-cell expansion and immune amplification in autoimmune settings through effects on antigen presentation and interleukin-2 signaling. Abbreviations: PAD4, peptidylarginine deiminase 4; DC, dendritic cell; IL-2, interleukin-2. The figure emphasizes that benefit, risk, and opportunity arise from the same PAD4-dependent pathways and are determined by immune context rather than by distinct molecular mechanisms.

Such risks suggest that the biological effects of PAD4 inhibition differ substantially across immune-mediated conditions. Rather than pursuing uniform inhibition across indications, PAD4 targeting may be most defensible where pathology is sustained by citrullination-dependent immune amplification and where controlled immune dampening is acceptable (**Figure 1**). Viewing PAD4 inhibition along a continuum that spans host defense, inflammation, and tolerance provides a coherent framework for aligning molecular mechanism with clinical intent and for identifying scenarios in which broader immune effects represent opportunity rather than liability.

## 5 Refining translational strategies for PAD4-associated outcomes

Framing PAD4 inhibition within a broader translational landscape highlights that many PAD4-associated pathological outcomes can be modulated through strategies that do not rely on direct enzyme inhibition. Interventions such as deoxyribonuclease treatment, modulation of renin–angiotensin axis (45, 46), targeting Gasdermin D-dependent inflammatory amplification (47, 48), or scavenging immunostimulatory NET components (49–51) illustrate that distinct points within PAD4-linked pathways can be therapeutically accessed. These approaches differ in timing, mechanism, and immune scope, and may be preferentially suited to specific disease contexts or stages. At the same time, they do not mimic PAD4 inhibition, but rather clarify the conditions under which direct targeting of citrullination is necessary to limit upstream immune activation. Considering PAD4 inhibitors alongside these complementary strategies helps refine indication selection, informs combination or sequential treatment designs, and situates PAD4 blockade as one element within a broader toolkit aimed at controlling NET-associated immune pathology.

Framing PAD4 inhibition within a broader translational landscape highlights the need for careful indication selection and biomarker-driven approaches to identify patients most likely to benefit. PAD4-targeting strategies are most appropriate in settings where citrullination-dependent pathology is sustained, such as those characterized by high NET burden or dysregulated neutrophil–platelet interactions. Biomarkers, including circulating NET components, citrullinated proteins, or platelet–neutrophil aggregates, can help pinpoint the right patient populations. Additionally, endpoint selection should go beyond measuring NET markers and focus on meaningful effects such as tissue injury, inflammatory resolution, and immune coordination. For autoimmune diseases, adaptive immune responses may require greater attention, while vascular and coagulation-related outcomes should take precedence in thrombo-inflammatory contexts. Aligning these factors with disease-specific mechanisms will be crucial for translating PAD4-related interventions into clinically relevant outcomes, balancing the therapeutic benefits of PAD4 blockade with its potential risks. .

## 6 Discussion

PAD4 inhibition emerges as a form of immune modulation that extends well beyond NETosis suppression. Across multiple disease settings, PAD4 activity contributes to inflammatory amplification through histone and non-histone citrullination, shaping innate effector functions, antigen presentation, and adaptive immune activation. In this light, many effects often described as off-target reflect predictable consequences of disrupting citrullination-dependent regulation rather than unintended pharmacological artefacts. The impact of PAD4 inhibition therefore depends strongly on disease context, timing, and exposure, with benefit arising where excessive immune activation dominates pathology and risk becoming prominent where intact host defense and tissue repair are required. These considerations underscore the limitations of evaluating PAD4 blockade using NET-centered endpoints alone and highlight the need for broader measures of immune coordination and function.

A more balanced view of PAD4 targeting follows when its molecular actions are aligned with clinical intent. In autoimmune disease, reduced citrullination may limit autoantigen generation, dampen T-cell priming, and constrain IL-2-dependent immune expansion, aligning immune dampening with therapeutic goals. In contrast, in infectious settings or during tissue repair, the same mechanisms may undermine protective responses. Recognizing this continuum allows PAD4 inhibition to be judged not as inherently beneficial or harmful, but as conditionally appropriate. Progress in this field will depend on improved selectivity, careful patient selection, and mechanism-aligned endpoints that reflect both efficacy and immune competence. Framing PAD4 blockade within this context-dependent model provides a rational path for translation and helps define where its broader immune effects represent acceptable trade-offs or genuine therapeutic opportunity.
